# Glioma targeted therapy: insight into future of molecular approaches

**DOI:** 10.1186/s12943-022-01513-z

**Published:** 2022-02-08

**Authors:** Keyang Yang, Zhijing Wu, Hao Zhang, Nan Zhang, Wantao Wu, Zeyu Wang, Ziyu Dai, Xun Zhang, Liyang Zhang, Yun Peng, Weijie Ye, Wenjing Zeng, Zhixiong Liu, Quan Cheng

**Affiliations:** 1 Department of Neurosurgery, Xiangya Hospital, Central South University, Changsha, China; 2 Xiangya School of Medicine, Central South University, Changsha, China; 3 National Clinical Research Center for Geriatric Disorders, Xiangya Hospital, Central South University, Changsha, China; 4 One-Third Lab, College of Bioinformatics Science and Technology, Harbin Medical University, Harbin, China; 5 Department of Oncology, Xiangya Hospital, Central South University, Changsha, China; 6 Department of Geriatrics, Xiangya Hospital, Central South University, Changsha, China; 7 Teaching and Research Section of Clinical Nursing, Xiangya Hospital of Central South University, Changsha, China; 8 Department of Clinical Pharmacology, Xiangya Hospital, Central South University, Changsha, China

## Abstract

Gliomas are the common type of brain tumors originating from glial cells. Epidemiologically, gliomas occur among all ages, more often seen in adults, which males are more susceptible than females. According to the fifth edition of the WHO Classification of Tumors of the Central Nervous System (WHO CNS5), standard of care and prognosis of gliomas can be dramatically different. Generally, circumscribed gliomas are usually benign and recommended to early complete resection, with chemotherapy if necessary. Diffuse gliomas and other high-grade gliomas according to their molecule subtype are slightly intractable, with necessity of chemotherapy. However, for glioblastoma, feasible resection followed by radiotherapy plus temozolomide chemotherapy define the current standard of care. Here, we discuss novel feasible or potential targets for treatment of gliomas, especially IDH-wild type glioblastoma. Classic targets such as the p53 and retinoblastoma (RB) pathway and epidermal growth factor receptor (EGFR) gene alteration have met failure due to complex regulatory network. There is ever-increasing interest in immunotherapy (immune checkpoint molecule, tumor associated macrophage, dendritic cell vaccine, CAR-T), tumor microenvironment, and combination of several efficacious methods. With many targeted therapy options emerging, biomarkers guiding the prescription of a particular targeted therapy are also attractive. More pre-clinical and clinical trials are urgently needed to explore and evaluate the feasibility of targeted therapy with the corresponding biomarkers for effective personalized treatment options.

## Introduction

The most common malignant primary brain tumor in adults is glioma. Based on the previous histological classification of gliomas from grade I to IV in WHO classification in 2016 [1], molecular biomarkers of different tumor types were updated in WHO CNS5 in 2021, bringing more benefits and meaningful instructions to clinic. Generally, gliomas are divided into circumscribed gliomas and diffuse gliomas, with the former one being benign and curable after complete surgical resection and the latter one being more malignant and unable to be cured following surgical resection alone. According to that the fifth-edition WHO Blue Books have emphasized, use of Arabic numerals for grading is recommended [2]. In addition, WHO CNS5 has proclaimed the importance of grading within tumor type. Thus 4 different families are divided: 1) Adult-type diffuse gliomas; 2) Pediatric-type diffuse low-grade gliomas; 3) Pediatric-type diffuse high-grade gliomas; and 4) Circumscribed astrocytic gliomas. Nevertheless, for convenience, here we review targeted therapy of gliomas still in an order of summing up different types of tumors in a certain grade. Thus overall, low-grade glioma (LGG) contains CNS WHO grades 1–2, while high-grade glioma (HGG) contains CNS WHO grades 3–4. LGG, accounting for 6% of CNS primary tumors in adult, usually presents more promising prognosis [3]. The more common CNS WHO grade 1 LGG in child have the best prognosis [4], while grade 2 LGG usually relapses and progresses to HGG [5]. Besides diffuse midline glioma H3 K27M-altered, glioblastoma (GBM) is the majority of WHO grade 4. GBM is one of the most lethal and prone to recurrence malignant solid tumor, accounting for 57% of all gliomas and 48% of primary CNS malignant tumors [6], with median survival time less than 2 years. Currently, standard magnetic resonance imaging could provide the most initial and sensitive diagnosis to GBM, while GBM diagnosed with such method have usually developed into advanced stage [7].

Both The 2016 WHO classification and WHO CNS5 have declared the mutational status of isocitrate dehydrogenase (IDH) should be considered regarding LGG, which emphasized IDH-wildtype (IDH-WT) as the critical biomarker of high-risk LGG since the molecular characteristics and clinical manifestations of IDH-WT LGG are similar to those of GBM. Analogously, glioblastomas with mutant IDH are characteristically similar to anaplastic astrocytoma (though nomenclature “anaplastic astrocytoma” is no longer included in WHO CNS5 classification), thus treatment of glioma much relying on molecular diagnosis and classification.

Except for IDH status, MGMT methylation is hitherto regarded as another significantly prognostic biomarker. Other markers in CNS5 are merely related to grade and further estimate prognosis, such as CDKN2A/B homozygous deletion in IDH-mutant astrocytoma, as well as 1p/19q co-deleted, TERT promoter mutation, EGFR amplification or mutation, and + 7/ − 10 copy number changes in IDH-wildtype diffuse astrocytoma [2]. Among those, only EGFRvIII mutation is unequivocally clinically instructional (see below). Recently researchers have also concentrated on mismatch repair (MMR) protein as a novel biomarker due to its high relevant association with tumor mutational burden (TMB) [8], though it appears MMR status in recurrence GBM is not a prognostic marker. Thus, treatment of glioma much relying on molecular biomarkers as criteria of diagnosis and classification.

Histologically, LGG tumor cells present nuclear atypia and increased mitotic activity, while GBM cells characteristically remain areas of microvascular proliferation, focal necrosis, or both [9]. Histological distinction does not make a difference to the current clinical treatment. However, the variety of molecular subtypes is often related to the treatment and prognosis of patients. Specifically, IDH-WT glioblastoma usually contains higher level of epidermal growth factor receptor (EGFR) amplification, TERT promoter mutation and PTEN deletion, etc. [10]. Meanwhile, patients with MGMT promoter methylation, observed in 30% to 50% of IDH-WT glioblastoma [11, 12], may present better prognosis and treatment response. Pediatric LGGs and those in adults are distinct in molecular characteristics, though similarities on histology exist a lot. Pediatric LGGs were thought to carry mutations of FGFR1 and BRAF, both concentrating on MAPK pathway, although recently in WHO CNS5 pediatric-type low-grade diffuse gliomas include Diffuse astrocytoma, MYB- or MYBL1-altered; Angiocentric glioma; Polymorphous low-grade neuroepithelial tumor of the young; and Diffuse LGG, MAPK pathway-altered. Adult LGGs are characterized by mutations of IDH1/2 and ATRX, with 1p/19q codeletion sometimes. TERT promoter mutation was also found in LGG, which has to do with oligodendroglioma. Moreover, Epithelioid glioblastoma, as a newly discovered GBM tissue subtype, often carries BRAF^V600E^ mutations. Since the consensus treatment currently is restricted to limited number of patients (as mentioned below) and most of gliomas failed to meet completely recovery, including either unexpected relapse or worse progression in LGGs and poor survival particularly in GBM, original insights into therapies are pressing. With regard to molecular heterogeneity, the importance of varying and adequate targeted therapy is self-evident in order to open up more possibility to treatment of gliomas, to say nothing of the fact that since the discovery of PD1/PDL1 awarded as Nobel Prize more and more immunotherapy options have been proposed and developing. Correspondingly, predictive biomarkers are strongly recommended to be identified for optimizing the efficacy of immunotherapy. For instance, MHC class I-negative glioma cells were found to be associated with inactivation and resistance to immunotherapy [8].

## Current standard of care

As discussed above, the prognosis of WHO grade 1 and 2 glioma is the most promising [13], whereas differing from classification of molecular phenotype. IDH mutation and 1p/19q codeletion tumor (corresponding to oligodendroglioma) has the best prognosis, followed by IDH mutation and 1p/19q intact tumor, and IDH wild type tumor [14]. Although it is previously thought that "wait and see" approach could be used safely and appropriately for LGGs, recent trials have found that surgical resection should be performed on patients as soon as possible to avoid subsequent malignant progression of the tumor and meanwhile to accurately identify the molecular subtypes of the tumor [15]. For high-risk LGG, due to the high possibility of recurrence, the standard of postoperative care is necessary, including 50-54 Gy local radiotherapy, followed by 6 cycles of adjuvant procarbazine or Lomustine or vincristine (PCV), in which Lomustine is usually selected, due to its respectively mild toxicity and blood–brain barrier limitations [14]. Recent decades, the replacement or combination of radiotherapy with chemotherapy and target therapy and individualized treatment for different patients have been gradually proposed [16–18]. For instance, for some patients with unresectable pediatric LGG, carboplatin and vincristine are regarded as standard treatment [19].

For GBM, a gross total resection, radiotherapy in the focal tumor area and concomitant Temozolomide (TMZ) chemotherapy and certain dose of radiotherapy should be taken as the standard treatment (Stupp treatment) [20, 21]. Tumor-treating field, as a novel strategy of care, improves progression-free survival (PFS) and overall survival (OS) [22], whereas not included in the current general consensus on GBM treatment. All glioblastomas will eventually progress or relapse, and there is no standard treatment for recurrent GBM (rGBM). Lomustine, another alkylating agent, most widely used in recurrent GBM and also in “control group” in the lately recurrent GBM randomized trial [23], is partially considered to be the standard choice for rGBM, but only effective in patients with MGMT methylation [6]. European association of neuro-oncology (EANO) proposed for patients with rGBM to continue using TMZ or Bevacizumab [20]. However, TMZ often produces drug resistance due to the non-methylated MGMT promoter in tumor cells of patients [24], and bevacizumab could only prolong the PFS of rGBM. Notably, the combined therapy presents better effect.

## Glioblastoma

### Alkylating agent and MGMT promoter methylation

Temozolomide, currently used in the standard treatment of GBM, is an alkylating agent that induces tumor cell death by alkylating DNA at multiple sites. O^6^-methylguanine DNA methyltransferase (MGMT) functions as a sort of repairing protein [25, 26], which is encoded by MGMT and could reverse this alkylation process by consuming itself. Hence, MGMT promoter methylation is a strong prognostic biomarker and brings benefits, at least theoretically, to patients treated with TMZ combined with radiotherapy [25, 27] (Table 1).

**Table 1:** Molecular targeted therapy of GBM

Reference& selected trials	Intervention	Design	Primary endpoint	Response	PFS	OS	Conclusions
EGFR							
Sepúlveda-Sánchez et al.2017	Dacomitinib	Non-randomized Phase II, Open label	PFS-6	EGFRamp/EGFRvIII- 1 CR, 1 PR EGFRamp/EGFRvIII + 1 PR	PFS-6(%) EGFRamp/EGFRvIII- 13.3 EGFRamp/EGFRvIII + 5.9 Median PFS(months) EGFRamp/EGFRvIII- 2.7 EGFRamp/EGFRvIII + 2.6	Median OS(months) EGFRamp/EGFRvIII- 7.8 EGFRamp/EGFRvIII + 6.7	Dacomitinib has a limited single-agent activity with EGFR amplification
Byeon et al. 2020	Gefitinib	Single-arm phase II, open label	ORR	1 PR and 2 SD	Median PFS 2.1 months	ND	Gefitinib is modestly active
Neyns et al. 2009	Cetuximab	Non-randomized phase II, Open label	RR	3 PR and 16 SD	PFS-6 7.3% Median PFS 1.9 months	OS-6 37.9% Median OS 5.06 months	Cetuximab is inactive with HGG
van den Bent et al. 2018	Depatuxizumab/ABT-414	Randomized phase II, open label	OS	ABT-414 plus TMZ 5 PR ABT-414 2 PR TMZ/CCNU 1 PR	Median PFS (months) ABT-414 plus TMZ 3 ABT-414 1.9 TMZ/CCNU 2.0	Median OS (months) ABT-414 plus TMZ 9.6 ABT-414 7.9 TMZ/CCNU 8.2	ABT-414 may be active in combination with TMZ
Schuster et al. 2015	Rindopepimut	Randomized phase II, Open label	PFS	ND	PFS-5.5 66% Median PFS 9.2 months	Median OS 21.8 months	Rindopepimut needs further study
Weller et al. 2017	Rindopepimut	Randomized phase III, Placebo-controlled	OS		Median PFS (months) rindopepimut 7.1 placebo 5.6	median OS (months) rindopepimut 20.1 placebo 20.0	Rindopepimut is inactive in newly diagnosed disease
PI3K/AKT/mTOR							
Chang et al. 2005	CCI-779	Non-randomized phase II	PFS-6	2 PR and 20 SD	ND	ND	CCI-779 is inactive
Wen et al. 2019	Buparlisib	Single arm phase II	PFS-6	none	PFS-6 8%	9.8 months	Buparlisib is inactive as single agent
Hainsworth et al. 2019	BKM120 with bevacizumab	Single arm Phase II	PFS	8 CR and 12 PR	PFS-6 36.5% Median PFS 4 months	OS(months) BEV-naïve 10.8 BEV 6.6	BKM120 is poorly tolerated and relatively inactive
Wick et al. 2016	Temsirolimus	Randomized phase II, open label	OS-12		Median PFS (months) temsirolimus 5.4 TMZ 6.0	OS-12 Temsirolimus 70% TMZ 72% Median OS (months) Temsirolimus 14.8 TMZ 16.0	mTORSer2448 phosphorylationmay be used for enrichment in further studies of mTOR inhibition
Ma et al. 2015	Everolimus	Single arm phase II	OS-12		median PFS 6.4 months	OS-12: 64% median OS: 15.8 MONTHS	Everolimus is not active in combination with TMZ/RT → TMZ
MET							
Wen et al. 2011	Rilotumumab	Single arm phase II	ORR	None	PFS (months) 10 mg/kg: 1.0 20 mg/kg: 1.0	OS (months) 10 mg/kg: 6.5 20 mg/kg: 5.4	Rilotumumab is inactive
Cloughesy et al. 2017	Onartuzumab	Randomized phase II, open label	PFS-6	onartuzumab plus bevacizumab 1 CR, 11 PR bevacizumab 3 CR, 11 PR	PFS-6 (months) onartuzumab plus bevacizumab 3.9 bevacizumab 2.9	OS (months) onartuzumab plus bevacizumab 8.8 bevacizumab 12.6	High tumor hepatocyte growth factor and lack of MGMT promoter methylation may predict benefit from MET inhibition
van den Bent et al. 2020	INC280(Capmatinib)	Non-randomized phase Ib/II open label	PFS-6	INC280 monotherapy 3 SD	ND	ND	INC280 is inactive as single agent
BRAF							
Kaley et al. 2018	Vemurafenib	Single arm phase II	ORR	None, 3 SD in 6 patients			BRAF inhibition in gial brain tumors deserves further study
FGFR							
Sharma et al. 2019	Dovitinib	2-arm Phase II Open label	PFS-6/TTP		PFS-6: 6%(± 4%) Median PFS 1.8 months	Median OS 5.6 months	Dovitinib is not active
Proteasome							
Friday et al. 2012	Bortezomib plus vorinostat	Single arm Phase II	PFS-6	1 PR	PFS-6: 0%	Median OS 3.2 months	No indication to further study this combination
Kong et al. 2018	boretezomib	Single arm Phase II	OS		Median PFS 6.2 months	Median OS 19.1 months	Bortezomib warrants further study
Huang et al. 2019	Disulfiram	Single arm Phase II Open label	ORR	6 SD	Median PFS 1.7 months	Median OS 7.1 months	Disulfiram has limited activity
CDK4/6 or CDKN2A/B or RB							
Taylor et al. 2018	Palbociclib	Single arm Phase II	PFS-6		Median PFS 5 weeks	Median OS 15 weeks	Palbociclib is inactive as single agent
Multi-kinase inhibition							
Wen et al. 2018	Cabozantinib	Single arm phase II	ORR	6 PR and 17 PR	PFS-6: 22.3% and 27.8%	Median OS 7.7 and 10.4 months	Cabozantinib is inactive as single agent
Cloughesy et al 2018	Cabozantinib	Single arm phase II	ORR	3 PR	PFS-6: 8.5%	Median OS 4.6 months	Cabozantinib is inactive as single agent
TGF-β							
Brandes et al. 2016	Galunisertib	Randomized phase II, Partially blinded	OS	Galunisertib plus lomustine 1 CR Galunisertib 2 PR Lomustine none	PFS(months) Galunisertib plus lomustine 1.8 Galunisertib 1.8 Lomustine 1.9	OS(months) Galunisertib plus lomustine 6.7 Galunisertib 8.0 Lomustine 7.5	Galunisertib is inactive
Bogdahn et al. 2011	Trabedersen	Randomized phase IIb, Open label Active controlled	Tumor control rate		Median survival 10 μM trabedersen 7.3 80 μM trabedersen 10.9	OS-24 10 μM trabedersen 20% 80 μM trabedersen 18%	Trabedersen needs further clinical development
PD-1							
Reardon et al. 2017	Nivolumab	Randomized phase III, Open label	OS	Nivolumab 12 responses Bevacizumab 36 responses	Median PFS (months) Nivolumab 1.5 Bevacizumab 3.5	OS (months) Nivolumab 9.8 Bevacizumab 10.0	Nivolumab may be active in patients with MGMT promotermethylated tumors who are not on steroids
Schalper et al. 2019	Nivolumab as neoadjuvant	Single arm Phase II			Median PFS 4.1 months	Median OS 7.3 months	Nivolumab is inactive as neoadjuvant

*PFS* progression-free survival, *PFS-6 6-month* PFS rate, *OS* overall survival, *OS-6 6-month* OS rate, *ORR* objective response rate, *RR* response rate, *ND* no data

Studies have confirmed that it’s better to initiate post-surgery TMZ chemotherapy within 6 months [28, 29]. Since the effect of TMZ combined with radiotherapy differs among patients, evaluating the status of MGMT promoter has been recognized for its significance. Though there is no such thing as international consensus on the best diagnostic method of measuring MGMT promoter [30], there have been various development to evaluate the level of MGMT methylation in patients with GBM [30–33]. There is occasionally no predictive relationship between the methylation level of MGMT promoter and the level of the corresponding protein. Clinical trial showed that TMZ might lead to recurrence of GBM with high expression of MGMT [34], and resistance to TMZ was presumed related to MGMT gene fusion or rearrangement mutation [35]. Thus, more effective target therapies are urgently needed.

Recently, animal models present that Bortezomib could increase the sensitivity of GBM to temozolomide by reducing MGMT mRNA and protein [36]. Newly discovered enhancer, namely K-M enhancer, increases MGMT expression thus inducing TMZ resistance despite of the hypermethylated MRMT promoter [37]. Therefore, the combination of TMZ and K-M enhancer inhibitors could be a potent treatment modality. Besides, Frenel et al. proved that the combination of folic acid, TMZ and radiotherapy in the treatment of unmethylated MGMT patients was feasible, suggesting the prospect of inducing MGMT methylation in GBM therapy (NCT01700569) [38].

### Tyrosine kinase receptor

#### Epidermal growth factor receptor (EGFR)

**Fig. 1:**
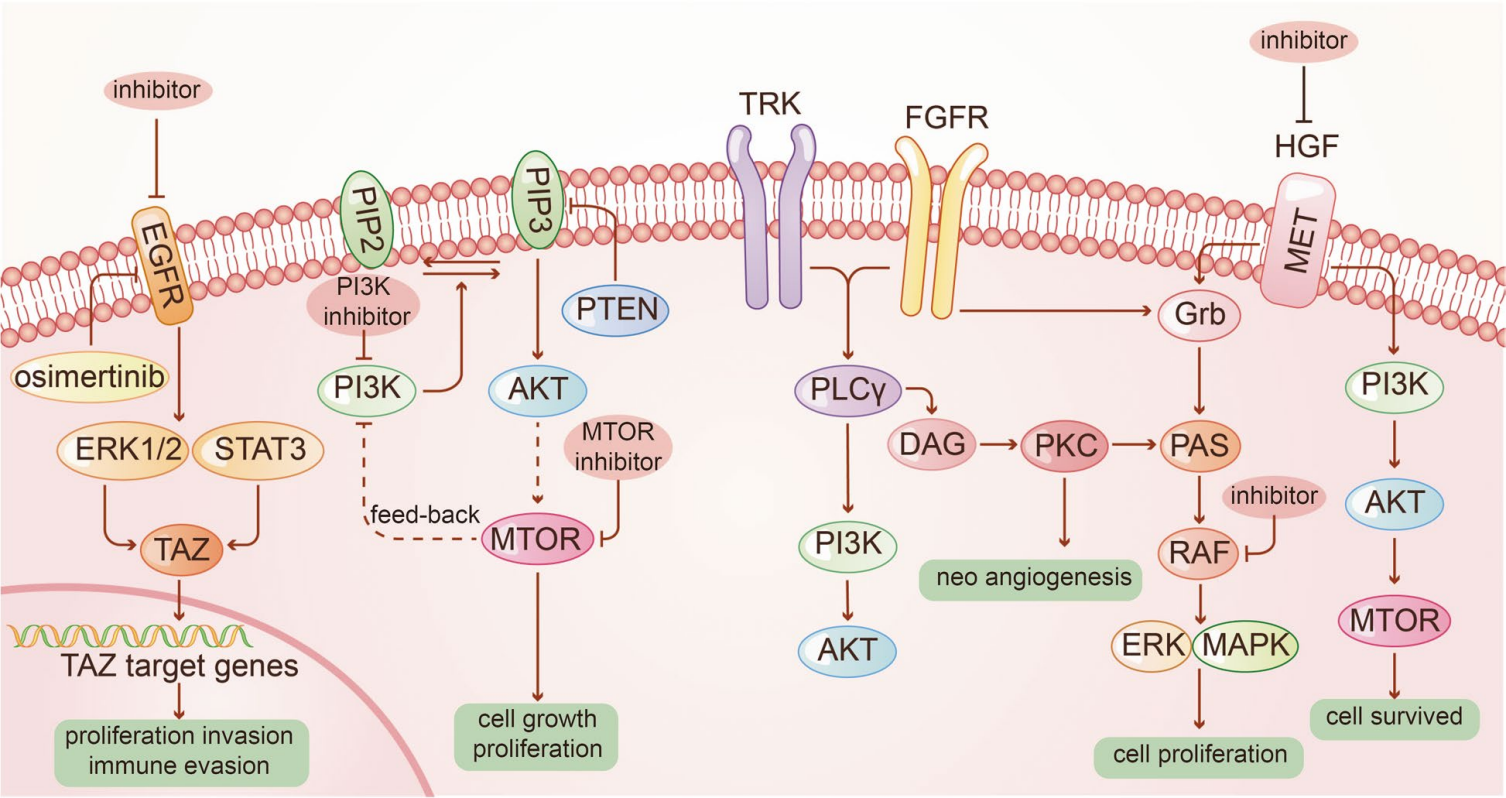
Tyrosine kinase receptor

Two measures are usually considered for treatment of GBM with EGFR as the target: one is to use EGFR inhibitors, and the other is to use antibodies, vaccines, CAR-T and other therapies to limit the content of EGFR (Fig. 1). EGFR is one of the most common oncogenic mutation sites in IDH-WT GBM [10], relevant to proliferation, migration and escape from apoptosis of tumor cells [39]. EGFR mutations occur in about 50% of all GBM samples, of which more than 40% are gene amplification, and the rest include gene mutations, rearrangements, splicing site changes, etc. [10, 40–43]. The most common gene mutation of EGFR is EGFRvIII (deletion of exons 2–7) [10], as a potential marker of treatment for GBM.

EGFR inhibitors, Gefitinib and Dacomitinib, are not so effective in the treatment of EGFR-amplification GBM patients (NCT01520870, NCT02447419) [44, 45], which is conjectured to be related to the blockage of blood–brain barrier (BBB). Osimertinib, as the third generation of inhibitor targeting EGFR in non-small-cell lung cancer (NSCLC), with better ability in BBB penetration, needs to be further verified before clinical application [46]. Recently, Osimertinib could not only inhibit EGFR-negative glioblastoma patient-derived xenograft (PDX), possibly via regulation of MAPK pathway [47], but also inhibit transcription factor EGFR-TAZ [48], providing a novel insight for drug reuse of EGFR-targeted inhibitors.

Antibodies of EGFR mostly failed in trials out of expectation [49, 50]. However, Nimotuzumab was more effective with patients carrying activated akt/mTOR [51]. Depatuxizumab (formerly ABT-806) showed limited efficacy, but Depatuxizumab mafodotin (formerly ABT-414), an antibody–drug conjugate using EGFR antibody as receptor-direction, seemed to be effective in recurrent GBM (rGBM) after standard treatment of TMZ [52, 53], but ineffective in newly-diagnosed GBM (NCT02573324) [54]. Novel methods are proposed to overcome the problem of BBB blockage with their feasibility to be verified before clinical application [55, 56]. Vaccination Rindopepimut combined with TMZ in rGBM patients carrying EGFR-VIII is relatively active (originally NCT00458601) [57], but it failed to present effectiveness in a phase III trial (NCT01480479) [58]. CAR-T therapy is still under phase I trial and demonstrates expected effect (NCT02209376) [59, 60].

#### PI3K/AKT/mTOR pathway

PI3K/mTOR is one of the most common mutation pathways in patients with IDH-WT GBM [10]. Activation of PI3K in GBM is mainly due to the mutation of phosphatase and tensin homolog on chromosome ten (PTEN) [61, 62]. Early in 2005, it was proved that the mTOR inhibitor temsirolimus was inactive as a single drug in rGBM [63]. A recent phase I clinical trial exploring the combined Temsirolimus and AKT inhibitor perifosine demonstrated disappointing results, but it was observed that patients had higher tolerance to Temsirolimus, which was speculated to be related to the use of corticosteroids in the experiment (NCT01051557) [64]. As a new PI3K pan-inhibitor, Buparlisib was also proved to be ineffective against rGBM in experiments, either as a single dose [65] or combined with carboplatin or Lomustine (NCT01339052, NCT01934361) [66]. Further research for an oral PI3K inhibitor, Bevacizumab with BKM120, was terminated due to low tolerance in patients (NCT01349660) [67]. Combination of Perifosine and Temsirolimus for rGBM is under test in an ongoing trial (NCT02238496).

The mTOR inhibitor Everolimus was not effective in patients with newly diagnosed MGMT promoter-unmethylated GBM, either used alone (NCT01019434) [68] or combined with radiotherapy or TMZ (NCT00553150) [69]. A recent phase I trial of Buparlisib combined with TMZ and radiotherapy in newly diagnosed GBM patients was interrupted due to adverse events and dose-limiting toxicities of Buparlisib, suggesting the deficiency of this combined treatment (NCT01473901) [70].

In short, PI3K pathway as a therapy target in GBM is often ineffective and followed with relatively low patient tolerance, which may be related to the complex molecular regulation of PI3K/AKT/mTOR. Some trials have shown that the tolerance of inhibitors will increase significantly under certain conditions that have not yet been explored, and it is possible to find ways to help patients tolerate higher doses in the future to ensure effects of targeted therapy. In addition, the current effect of PI3K inhibitors combined with other treatments is not ideal, which more combination strategies should be explored in the future.

#### MET

MET gene encodes hepatocyte growth factor receptor (also known as scatter factor), which is thought to play an important role in the migration, invasion, drug resistance and recurrence of glioma cells, especially in radiation resistance, inhibition of angiogenesis and hypoxia [71, 72]. About 30% GBM patients are charactered by MET hyper-expression [73]. The use of AMG102 (Rilotumumab) antibody alone had no effect on inhibiting the progression of GBM [74]. A clinical trial of combined antibody Onartuzumab and antivascular drugs confirmed that there was no significant benefit for rGBM patients but those with high expression of HGF (NCT01632228) [75]. Cabozantinib, an inhibitor of MET, whether in combination with antiangiogenic drugs or not, was mildly active in patients with rGBM (NCT00704288) [76, 77]. Combined Buparlisib and MET inhibitor Capmatinib failed to prolong the survival of PTEN-loss recurrence GBM patients (NCT01870726) [78]. Since mutations in c-MET often lead to drug resistance in GBM patients, influencing the efficacy of PI3K targeted therapy, the combination of MET inhibitors and PI3K inhibitors can be considered in follow-up trials.

#### Fibroblast growth factor receptor (FGFR)

FGFR is widely expressed in GBM, but its therapeutic value may be limited to a small number of patients with FGFR-TACC fusion [79]. One case with stable disease and one case with partial response were reported in 2 FGFR3-TACC3-positive rGBM patients treated with oral pan-FGFR kinase inhibitor Erdafitinib [80]. Similarly, only partial response in FGFR3-TACC3 positive GBM patients treated with this inhibitor was reported in another phase I trial (NCT01703481) [81]. In recent trials, the use of Dovitinib, an oral inhibitor of FGFR and VEGFR, whether combined with anti-vascular therapy or not, was ineffective in prolonging survival in patients (NCT01753713) [82].

#### BRAF mutation

BRAF, a member of Raf kinase family, participates in activation of Mek/Erk signaling pathway and promotes cell proliferation [83]. Mutations of BRAF, particularly BRAFV600E missense mutation, are observed in multiple types of cancer and have been proved to be a reliable target [84–87]. Although BRAF mutation was observed in several glioma subtypes, it was rare in high grade gliomas including GBM [88]. The low mutation rate of BRAF in GBM limited the therapeutic effect [89–92].

#### Neurotrophic tyrosine receptor kinases (NTRK)

NTRK is encoded by three different genes, namely NTRK1, NTRK2 and NTRK3. The genomic rearrangement of NTRK gene leads to gene fusion [93], which may trigger the activation of carcinogenic TRK signaling pathway. The incidence of NTRK gene fusion seems to be quite rare in glioblastoma [94]. An adult GBM patient with IDH-WT and NTRK2 rearrangement was treated with Larotrectinib and Entrectinib successively, showed a robust but temporary response. Re-biopsy after disease progression showed that the tumor cells carrying rearranged NTRK2 were eliminated and the tumor cells with amplification of PDGFRA survived [95]. Larotrectinib was also used in a female patient with infantile GBM, and the curative effect was significant [96]. Entrectinib was also effective in the treatment of infantile GBM [97], indicating the potential therapeutic value and diagnostic value of NTRK fusion for GBM.

### Cell cycle control and apoptosis regulating pathways

#### The retinoblastoma (pRB) pathway

**Fig. 2:**
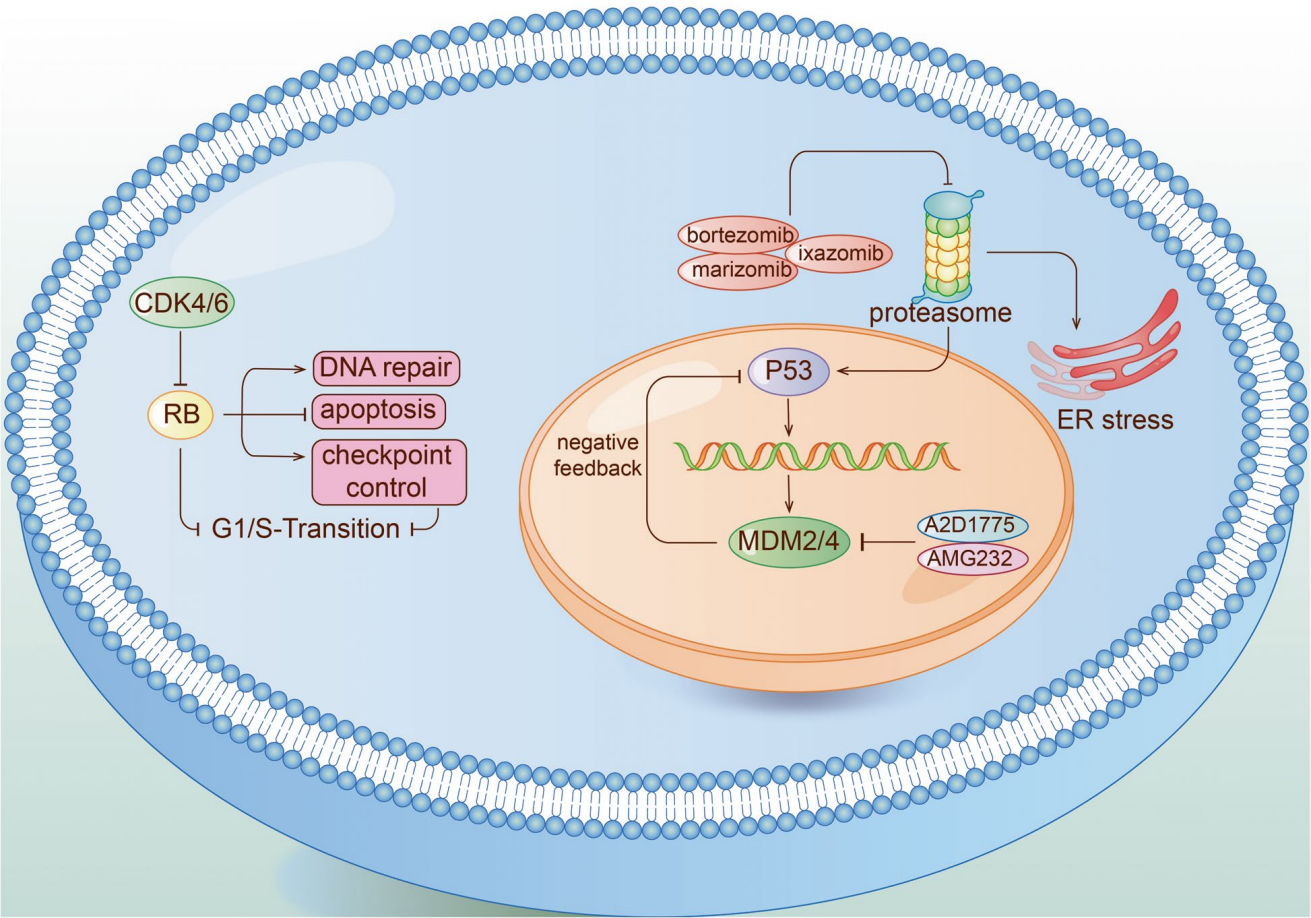
Cell cycle control and apoptosis regulating pathways

In most IDH wild-type GBM, the cell cycle control of pRB pathway is alternated due to homozygous deletion of CDKN2A/B, amplification of CDK4/6, and change of RB1 gene (Fig. 2). Challenging abstacles appears when applying pRB pathway as clinical target, due to the extensive existence of this pathway in normal cells [98]. CDK4/6 inhibitor Palbociclib for GBM was disappointing in a phase II trial (NCT01227434) [99]. Ribociclib as a single agent was also ineffective (NCT02933736) [100, 101]. SPH3643, as a newly-discovered inhibitor of CDK4/6 has not been tested in clinical trials, but its BBB permeability may indicate better clinical benefits than Palbpciclib [102]. TG02 is a multi-CDK inhibitor mainly targeting CDK9 rather than CDK4/6, currently being tested in clinical trials for rGBM and newly diagnosed GBM (NCT02942264, NCT03224104).

#### The p53 pathway

TP53, as a tumor suppressor, has been deeply elucidated in GBM. Given the key function of p53 in blocking cells in G0/1 and in inducing apoptosis in response to genotoxic stress [103, 104], how to restore the function of p53 has been widely studied. Although drugs for promoting the refolding of mutant proteins into wild-type conformations have not been successful, continuous efforts have been put in inhibiting the negative regulatory proteins of p53, MDM2 and MDM4, which aims at neutralizing defective MDM2 and MDM4 produced by amplification of MDM2 and MDM4 gene in GBM patients [105, 106]. The MDM2 inhibitor AMG 232 suppressed tumor progression in the course of the trial (NCT01723020) [107]. AZD1775, an inhibitor of Wee1 kinase, showed better brain tumor penetration but further trials are needed to prove its curative effect [108].

#### TERT promoter mutation

TERT promoter mutation is one of the most common molecular markers in IDH wild-type GBM [10, 109]. Two hot spots of TERT mutation produce new E-twenty-six transcription factors binding sites and increase TERT transcription, thus increasing TERT activity [110].

It was previously thought that the effect of MGMT promoter methylation on chemotherapy sensitivity and prognosis may be different in tumors with and without telomerase reverse transcriptase (TERT) promoter mutation [111]. However, recent studies have found that when patients with MGMT promoter methylation are treated with standard TMZ chemotherapy, TERT is likely to exert a positive effect [112].

TERT promoter mutation has not yet become the main pharmacological target for tumor therapy. Tubulin polymerization inhibitor Eribulin exerts TERT inhibitory activity in GBM model, which proves the rationality of its clinical application [113]. The mutation of TERT promoter creates a binding site for GABP transcription factor complex. Down-regulation of GABPB1L, an isomer of a subunit of GABP, could significantly improve the survival rates when combined with TMZ in GBM model, shedding light on the significance of finding its inhibitor [114]. Bases editing by CRISPR/Cas9 could correct TERT mutation and reduce the binding activity of ETS transcription factors to slow down tumor growth [115], but the prospect of gene therapy in clinic is still open to question.

#### Proteasome

As the vital mediator of intracellular degradation of useless or/and toxic proteins [116], proteasome promotes apoptosis by regulating p53 and ER stress, which critically regulates cell cycle and affects drug resistance of tumor cells [117]. Currently, bortezomib, Ixazomib, and Marizomib have been the clinically approved proteasome inhibitors.

Bortezomib combined with Vorinostat, a histone deacetylase inhibitor, was ineffective in rGBM (NCT00641706) [118], while Bortezomib combined with standard radiotherapy was well-tolerated and presented promising survival rates (NCT00998010) [119]. Marizomib combined with TMZ is ongoing a phase III trial (NCT03345095), and Marizomib combined with Bevacizumab is ongoing a phase I/II trial (NCT02330562). Ixazomib has distinct permeability to tumor tissues preclinically with its efficacy trials to be further verified [120].

Disulfiram not only restricts the proteasome from peripheral blood cells to a certain extent, but also has favorable BBB penetration ability and better drug resistance to exert its anti-tumor effects [121] in newly-diagnosed GBM and rGBM models. However, a phase II trial reported that Disulfiram was limited in sensitizing TMZ (NCT03034135) [122].

### Microenvironmental targets—Angiogenesis

#### Vascular Endothelial Growth Factor (VEGF)

**Fig. 3:**
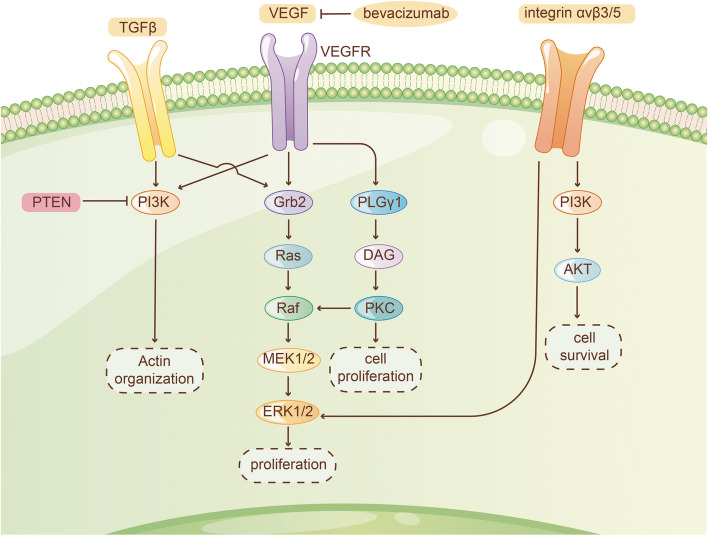
Microenvironmental targets

GBM is characterized by the abnormality in vascular proliferation (Fig. 3). VEGF is highly expressed in glioblastoma and promotes the abnormal proliferation of tumor blood vessels. VEGFR1 and VEGFR2 signaling pathways are suggested as the critical factor of tumor survival in GBM [123]. Hypothetically, vascular normalization could increase tumor blood perfusion and help improve patient survival (NCT00035656) [124].

Bevacizumab, a humanized monoclonal antibody against the VEGF-A ligand, binds to endothelial cells and inhibits angiogenesis [125]. In Phase II clinical non-controlled trials, Bevacizumab presents significant biological activity, anti-glioma activity, high radiation response rate (RR), high overall survival (OS) and 6 months PFS(PFS-6) in newly diagnosed and rGBM [126–128]. In Phase III clinical trials, Bevacizumab could also significantly improve PFS (NCT00884741) [129]. However, it did not improve OS even with the adjuvant chemoradiotherapy or lomustine and was reported with high frequency of adverse events (NCT00943826, NCT01290939) [23, 129, 130], and the quality of life raised, declined or remained the same which reached opposite results in different trials [129, 130]. IDH1-wildtype GBM patients exhibited prolonged OS after receiving Bevacizumab therapy (NCT00943826) [131]. Bevacizumab could develop drug resistance within months. The efficacy of multi-kinase inhibitor Ponatinib in GBM patients is very limited in Bevacizumab-refractory GBM (NCT02478164) [132]. Also, patients who progress on VEGF R-TKi with Bevacizumab only benefit modestly [133]. The latest research showed that the response to Bevacizumab in some patients might correlate with antibody dependent cytotoxicity(ADCC) [134].

Bevacizumab plus Temozolomide exhibits great efficacy and tolerance [135]. In some trials, Bevacizumab plus Trebananib had less effect than single agent (NCT01609790, NCT01609790) [136, 137]. Bevacizumab plus erlotinib or metronomic Etoposide had a similar effect to Bevacizumab monotherapy and Etoposide showed a greater toxicity (NCT00671970, NCT00612430) [138, 139]. The efficacy of small dose Bevacizumab plus Lomustine was not improved [140]. Bevacizumab combined with CCNU radiotherapy significantly improved PFS in IGS-18 GBM [141]. Although Bevacizumab plus Rilotumumab showed 3–4 months improvement in median OS over single agents in rGBM, PFS was not increased (NCT01113398) [142]. Administration of concurrent Bevacizumab and Erlotinib presented significantly higher RR and PFS-6 [143].

Other VEGF inhibitor like Cediranib, a pan-VEGF receptor tyrosine kinase inhibitor, showed significant efficacy and PFS-6 in phase II clinical trial of rGBM (NCT00305656) [144]. In newly-diagnosed GBM patients, Cediranib promoted the blood perfusion and prolonged the OS (NCT00662506) [145]. Cediranib also declined the tumor-associated angiogenic brain edema (NCT00254943) [146]. However, it failed to prolong the PFS in phase III clinical trial of rGBM (NCT00777153) [147].

Pazopanib and Tivozanib have in situ bioactivity and similar tolerance to other anti-VEGF drugs, but they failed to prolong PFS and OS in phase II trials ((NCT00459381, NCT01846871, NCT00350727)) [148–150]. Phase I trial of Aflibercept showed great toxic side effect to rGBM while the efficacy is very limited [151]. A phase II trial suggested that Aflibercept binds to VEGF with a greater affinity than Bevacizumab, whereas, without greater efficacy [152]. Axitinib, a tyrosine kinase inhibitor (TKI) against VEGFR-1, 2 and 3, could be a potential combination partner with immunotherapy (NCT01562197) [153]. Other inhibitors like Aflibercept could also down-regulate the activity of VEGF and needs to be further studied (NCT00369590) [154].

#### Integrin

Integrins are a family of 24 heterodimeric cell surface receptors that participate in signal transduction involved in many cellular processes. They also mediate cellular communication within the extracellular matrix during adhesion, motility, migration, invasion and angiogenesis. Integrins αvβ3 and αvβ5 are highly expressed in endothelial cells and identified as preclinical therapeutic targets in GBM [155, 156].

Cilengitide is a selective integrin inhibitor targeting αvβ3 and αvβ5, which its combination with Cediranib had a great tolerance to rGBM patients in a phase I trial (NCT00979862) [157]. In a phase II trial, Cilengitide has a moderate efficacy which could be transported and accumulated in rGBM cell through binding with avβ3 and αvβ5 [158, 159]. TMZ/RT-TMZ plus Cilengitide with great tolerance and efficacy could not improve invasiveness or recurrent rate of newly-diagnosed GBM (NCT00813943) [160, 161]. In GBM patients with MGMT promoter methylation, Cilengitide had good performance as adjuvant administration with standard treatment (NCT00689221, NCT00689221) [162, 163]. But Cilengitide failed to reach the primary endpoint in non- methylated patients (NCT01124240) [164]. In phase I and II trials, Cilengitide was proved incapable of being the monotherapy in children with GBM [165, 166]. A phase III trial showed limitation on the efficacy of Cilengitide (NCT00689221) [167]. Although Cilengitide has not exhibited remarkable potential as monotherapy, integrins remain to be the important target.

#### Transforming growth factor (TGF)-β

The TGF-β protein family has complex functions in a wide range of regulatory pathways [168, 169], among which TGFβ2 is a T cell suppressor in tumor microenvironment of GBM [170] and is expressed in about 90% of GBM tumor cells. However, although TGFβ1/2 inhibitors have been used in treatment of other cancers, they are still difficult to be used as GBM clinical treatment targets.

As a TGF-β receptor(R)1 kinase inhibitor, Galunisertib was ineffective with combined Lumostine (NCT01582269) [171]. TGF-β2-specific antisense oligonucleotides, Trabedersen was effective in particular to patients whose KPS are above 80% and age are under 55, but generally the therapy efficacy was far from expectations (NCT00431561) [172]. Similarly, antisense oligonucleotides, namely ISTH1047 and ISTH0047, exhibit anti-tumor properties and can be further tested in clinical trials [173].

Gene therapy focusing on hematopoietic stem cell (HSC) that expresses TGF-β blocking peptides enhanced the sensitivity of GBM to chemotherapy in animal model [174]. Given that, further clinical treatment can be considered. Recent trials have also pointed out that TGF-β is related to TMZ resistance and MGMT expression [175]. Therefore, the combination of TMZ and TGF-β inhibitors are promising.

### Immunotherapy

#### Programmed cell death protein (PD)-1

**Fig. 4:**
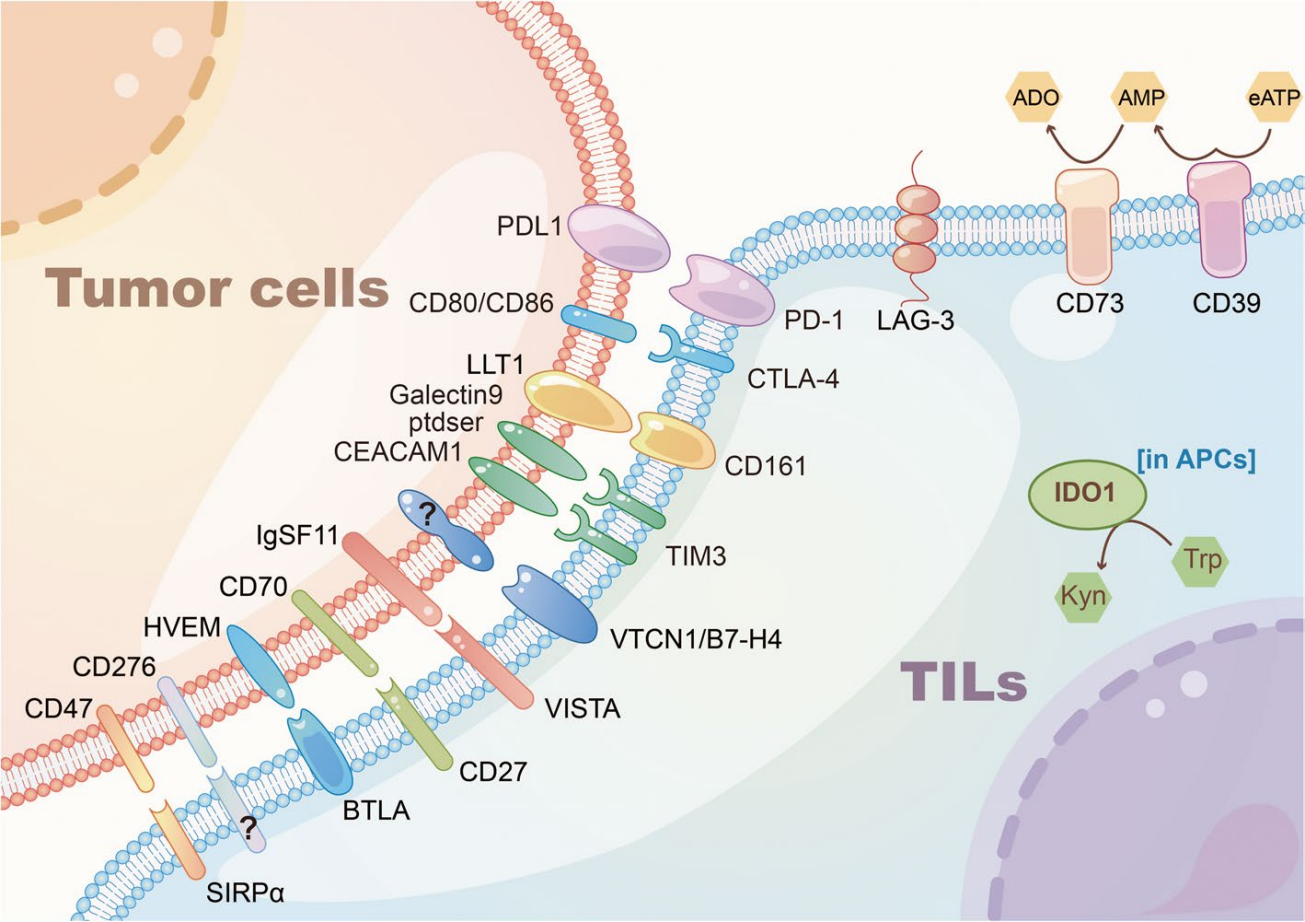
Immunotherapy targets

One strategy of cancer immunotherapy is to prevent the interaction between PD-1 ligand (PD-L1) on tumor cells or host cells and PD-1 on T cells (Fig. 4). Pembrolizumab, an antibody that blocks PD-1, has poor efficacy in previous treatments of GBM [176], except in cases with specific mismatch repair defects [177–179]. Thus, mismatch repair defects are expected to be a novel biomarker of targeting PD-1/PD-L1, with classic markers as TMB, tumor infiltrating lymphocyte (TIL) and microsatellite instability (MSI) [180–182]. However, standard therapy with neoadjuvant Pembrolizumab demonstrates significant survival benefits [183].

In a randomized clinical trial, Nivolumab combined with Bevacizumab [184] and Nivolumab combined with chemoradiotherapy in newly-diagnosed GBM patients with MGMT promoter unmethylation (CHECKMATE 498, NCT02617589) were both ineffective. Preclinic animal models confirmed that PD-1 blockade could reverse peripheral T cell exhaustion induced by TMZ but could not affect tumor infiltrating lymphocytes [185], which partly accounts for its ineffectiveness. The phase II trial of Nivolumab as neoadjuvant therapy also failed to show clinical benefits (NCT02550249) [186].

#### Lymphocyte activation gene 3 (LAG-3)

LAG-3, mainly found on activated immune cells [187, 188], leads to immune escape of tumor cells similar to that of PD-1 [189]. In tumor tissues, LAG-3 is usually expressed in T cells with lost functions, called exhausted T cells. Thus, inhibitor of LAG-3 become an attractive immune-modulating agent alone or in combination with other immune checkpoint inhibitors [190, 191].

In GBM, LAG-3 rather than PD-1 is co-expressed with CD8A [192], indicating that LAG-3 targeted therapy in GBM with abundant CD8 + T cells infiltration may be promising. A phase I trial (NCT02658981) of the LAG-3 antibody BMS-986016 is ongoing.

#### Cytotoxic T-lymphocyte–associated antigen 4 (CTLA-4)

CTLA-4 shares common receptors CD80/CD86 with CD28, and binding of CTLA-4 to those receptors are antagonistic to T cell activation and proliferation [193, 194]. CTLA-4 antibody Ipilimumab is the first clinically approved immune checkpoint inhibitor, in addition to Tremelimumab, etc. [182].

Combined anti-CTLA-4 and anti-PD-1/PD-L1 therapy is active in multiple kinds of cancers. Intracerebral injection of Ipilimumab plus Nivolumab is safe and feasible, and has a certain effect for rGBM (NCT03233152) [195]. Other trials for combined anti-PD-I and anti-CTLA-4 therapy are ongoing (NCT04323046, NCT04606316). The effectiveness of CTLA-4/PD-1/IDO triple therapy is also confirmed in animal model [196], further ensuring the prospect of combined immune therapy. The clinical benefits of Ipilimumab combined with standard chemoradiotherapy are observed in a phase II clinical trial (ISRCTN84434175) [197]. Intratumorally injection of IL-12 combined with systemic administration of CTLA-4 antibody is also effective in animal model [198], providing the robust basis for further clinical research.

Vaccination aimed at enhancing anti-glioma immunity by injecting autologous glioma cells mixed with GM-K562 cells that are inactivated via irradiation has been proved safety and modestly feasible [199]. Meanwhile, CAR-T therapy combined with Ipilimumab for the treatment of rGBM is also in the preliminary stage (NCT04003649).

#### CD73

CD73, an extracellular nucleotidase, catalyzes the production of adenosine from extracellular AMP [200], which exerts an immunosuppressive effect on GBM and induces drug resistance of vincristine presumedly via regulating multiple drug associated protein 1 (Mrp1) [201, 202].

Nasal administration of cationic nanoemulsion mixed with CD73-siRNA presented promising anti-CD73 effect in GBM model [203]. This anti-CD73 effect promotes, subsequently, alteration in tumor microenvironment and suppressing the tumor proliferation [204]. Whereas the feasibility of cationic nanoemulsion to clinic is under studied. Anti-PD-1 combined with anti-CTLA-4 showed a favorable effect in CD73-deficient GBM model [205]. Currently, there are still few clinical trials of CD73 inhibitors with GBM. Although CD73 inhibitor AB680 is discovered, it has not been used in treatment of GBM [206].

#### CD161

CD161, as the marker of GBM tumor infiltrating lymphocytes, is widely expressed on the plasma membrane surface of NK cells, CD8 + and CD4 + T cells [207], binding to the ligand CLEC2/Lectin like transcript-1(LLT1) mainly expressed in GBM myeloid cells [208]. Previous studies have confirmed that CD161 directly interacts with intracellular acid sphingomyelinase to regulate Akt signaling pathway and then inhibits activity of NK cell. Notably, CD161 simultaneously exerts the property of stimulating immunity and inhibiting immunity: when CD161 binds to LLT1 and is co-stimulated by CD3, the expression of TNF-α in T cells is promoted, when CD161 is stimulated on CD8 T cells alone, the expression of TNF-α in T cells is inhibited [209]. CD161 has been regarded as a critical regulator for immunosuppression in GBM [210]. So far, inhibition of CD161 is proved with enhanced anti-tumor effect of T cells in preclinic model.

#### IDO1

Indoleamine 2,3-dioxygenase 1 (IDO1), an Trp dioxygenase extensively detected in mammalian tissues except the liver [211–213], usually degrades Trp into L- kynurenine that subsequently activates aryl hydrocarbon receptor (AhR) via the Kyn–AhR–AQP4 signaling pathway, which promotes cell motility and increases malignancy of gliomas. Furthermore, IDO1 is regarded as a significant mediator in immunotolerance and immunosuppression of cancer via its non-enzyme activity, though the potential mechanisms remains to be fully elucidated [214]. Thus IDO1 is becoming an attractive target of immunotherapy in grade IV gliomas, especially GBM.

1-methyl-l-tryptophan (1-MT, also known as Indoximod), the inhibitor of IDO1, was effective with combined TMZ in animal models of malignant gliomas (exclusively corresponding to WHO grade IV gliomas) [215], hence several phase I/II trails exploring the safety and efficacy of combined 1-MT and chemotherapy in both pediatric and adult patients with gliomas are ongoing. (NCT02052648, NCT02502708, NCT04049669).

Given that some trials indicated inactivity of IDO1 inhibitor as a single agent, combination of IDO1 inhibitor and PD-1/PD-L1, CTLA-4 blockade [216], or other treatments such as anti-angiogenesis is proposed. Also, Erik Ladomersky et al. discovered that older patients with GBM experienced an age-related immunosuppression hypothetically resulted from the increase of IDO accumulation in elderly brain [217]. Since immunosuppression could be induced by Trp depletion and Kyn activation that are initiated by three enzymes namely IDO1, IDO2 and TDO while IDO1 and TDO are more vital than IDO2 in pathologic grade of gliomas [214], the application of IDO1 and TDO inhibitors simultaneously, excluding IDO2, is advised. Additionally, according to the fact that compared to the physiological situation systemic Kyn decreases while intratumoral Kyn increases in GBM patients, exploration on Kyn pathway modulation is still in its infancy [218]. Nevertheless, recent study has found that IDO induces the expression of complement factor H (CFH) and its isoform, factor H like protein 1 (FHL-1) independent of its enzymatic activity, which contributes to poor survival of GBM patients [219]. This finding would help explore the novel targets of IDO inhibition.

#### Hepatitis A virus cellular receptor 2 (HAVCR2)

HAVCR2 is a specific cell surface protein encoded by homonymous gene. It is also named T cell immunoglobulin and mucin-domain containing-3 (TIM3) [220] that belongs to immunoglobulin superfamily. TIM3 participates in regulation of macrophage, induction of immunological tolerance, inhibition of Th1-mediated auto- and alloimmune responses, which becomes a promising target for immunotherapy.

Generally, TIM3 interacts with HLA-B-associated transcript 3 (BAT3) and subsequently recruits kinase LCK to maintain T cell activation. However, Galectin 9, mainly found in tumor cells and antigen-presenting cells, binds to TIM3 that phosphorylates intracellular domain of TIM3 and recruits kinase FYN, contributing to apoptotic and anergic T cells. So far, various ligands of TIM3 have been discovered, such as carcinoembryonic antigen-related cell adhesion molecule 1 (CEACAM1), phosphatidylserine (PtdSer), and high mobility group protein B1 (HMGB1) [221].

Almost all anti-TIM3 antibodies presenting antitumor activity are designed to interfere with the binding between TIM3 and CEACAM1, PtdSer [222]. In particular, TIM3 is widely expressed in GBM and IDH-WT gliomas, regulating inflammatory activation especially after anti-PD-1 therapy [223, 224]. Thus, combined anti-TIM3 inhibitor and other immunotherapy is getting promising. Combination of anti-TIM3 therapy, anti-PD-1 therapy, and radiotherapy in animal models has reported promising efficacy [225]. Besides, MBG-453 is an antibody against TIM3 in an ongoing phase I trial (NCT03961971).

#### V-set domain containing T cell activation inhibitor 1 (VTCN1)

Commonly known as B7-H4, VTCN1 is a highly revolutionarily conserved type-I transmembrane protein [226]. Interaction of B7-H4 with the unknown receptors has the capability of negatively regulating activity of T cells. Moreover, VTCN1 not only inhibits production of cytokines but also arrests cell cycle in G0/Gi phase [227]. B7-H4 is found in a wide range of lymphocytes, including NK cells, T cells, and cancer cells, including partial glioma cells [228–230]

Previous study has declared that patient with high B7-H4 expression specifically experienced deficiency in tumor-infiltrating lymphocytes, suggesting its critical role in immunosuppression [230]. However, antibodies or inhibitors against B7-H4 remain to be developed for gliomas.

#### V-domain immunoglobulin suppressor of T cell activation (VISTA)

VISTA has been initially recognized for its significant role in immunosuppression [231]. VISTA complicatedly and conversely functions as both ligand and receptor in the negative or positive regulation of cancer immunity. Notably, several recent studies using anti-VISTA methods, VISTA-deficient models or computer simulation have confirmed its suppressive role and upregulation of immune response [232, 233].

One of the known ligands of VITSA, the immunoglobulin superfamily 11 gene (IgSF11), is found with elevated expression particularly in high grade glioma (also named HGG) and correlates with worse prognosis [234], suggesting the potential prognostic value of VISTA and IgSF11. However, clinical trials on targeting VISTA alone or as adjuvant therapy with gliomas are still scarce to date。

#### CD27/CD70

As a member of the tumor necrosis factor receptor (TNFR) superfamily widely expressed on resting T cells [235], NK cells [236], and memory B cells [237], CD27 interacts with its ligand CD70 to stimulate T cell activity [238]. Nevertheless, early finding also showed that CD27 correlates with apoptosis of CD27-bearing cells [239], indicating its two-sided role in regulating immune response [240].

CD70 is overexpressed in primary and recurrent glioma cells in contrast to normal tissue and lymphocytes, and is associated with poor survival. Thus, CD70 on tumor cells is proposed to induce T cell (especially CD8 + T cell) exhaustion or apoptosis and activate regulatory T cells (Tregs) to mediate immunosuppression [241–244]. Since CD27 stimulates T cell activity, and also induces cytotoxicity and then apoptosis of CD27-bearing lymphocytes under pathological cases, the combined CD27 agonist and CD70 inhibitor is promising.

Previous study has shown that agonist anti-CD27 mAbis capable of recruiting CD8 + T cells and promoting anti-tumor response in animal model [245]. CD27 agonist, Varlilumab, has been tested in combination with anti-PD-1 therapy in a phase I/II trial (NCT02335918), with the results to be reported. Other trials exploring anti-CD27 inhibitor as neoadjuvant or in combination with other immunotherapy options are ongoing (NCT02924038, NCT03688178). CAR-T cells target at CD70 alone or at both CD70 and B7-H3 present promising perspective, however not applied to clinical trials hitherto [244, 246].

#### B and T lymphocyte attenuator (BTLA)

As a member of CD28 superfamily, BTLA shares a similar molecule structure with PD-1 and CTLA-4. Functionally, as the only identified ligand of BTLA, herpes virus entry mediator (HVEM) [247, 248] interacts with BTLA to negatively regulate activity and proliferation of T cells.

It has been confirmed that BTLA influences T cell signaling through SHP1/2, similar to that of PD-1. But compared with PD-1 that prefers to recruit SHP2, BTLA mainly recruits SHP1. Surprisingly, under SHP1/2 deficient condition, BTLA still presents inhibitory effect of cell proliferation and cytokine production in primary T cells. Thus, combination of PD-1 blockade and BTLA blockade is promising and attractive, with some potent signaling pathways by which BTLA and PD-1 inhibit activity of T cell remain to be further elucidated [249, 250]. Preclinic model proved that anti-BTLA and anti-PD-1 immunotherapy mainly promotes the activation of CD4 + T cells, CD8 + T cells and the secretion of IFN-γ that correlates with favorable survival [251]. However, there is still a long way to go before this combination therapy could be applied clinically.

#### CD39

CD39 predicts worse survival for GBM and anaplastic astrocytoma patients [252]. As an ecto-enzyme hydrolyzing extracellular ATP (eATP) into AMP, CD39 and CD73 sequentially convert eATP to immunosuppressive adenosine (ADO) in the tumor microenvironment (TME), as one portion of the ATP–adenosine axis [253]. In the TME, eATP is released and accumulated due to hypoxia, subsequentially to stimulate inflammation activity or be converted to ADO by CD39 and CD73. ADO could be also generated by sequential catabolism of NAD + by CD38-CD203a-CD73 [253], and by alkaline phosphatase independently [254]. High level of extracellular ADO is beneficial to suppress immune response via ADO binding to low-affinity adenosine receptor namely A_2A_ and A_2B_ that are broadly expressed on lymphocytes and myeloid cells [255]. CD73 is proved to be preferentially expressed on glioma cells and has the synergetic effect with CD39 expressed on tumor-infiltration T cells on inducing immunosuppression [256, 257].

Researchers have revealed that CD39 expressed on tumor-associated macrophage could contribute to dysfunction of CD8 + T cell [258]. However, inhibitor for the adenosine pathway failed in preclinic model, with presumed explanation such as irreversible exhaustion of T cells [257], or different affinity and distribution of adenosine receptors on glioma cells or lymphocytes. Given that, the specific mechanism involved in the modulation of ADO in tumor microenvironment of glioma needs to be further revealed.

#### CD276

CD276, also known as B7-H3, is one of the B7 ligand family. CD276 is believed to provide a negative costimulatory signal both on donor T cells and host cells during transplantation [259, 260]. CD276 expressed on antigen-presenting cells also conveys an immunosuppression signal. Besides, CD276 is also detected on DCs, NK cells and epithelial cells.

CD276 is extensively overexpressed on tumor cells and tumor vasculature [261, 262], and serves as a hazardous marker in GBM as it mediates immunosuppression via inhibiting activity of NK cells, inducing invasion and differentiation of tumor cells [263–265]. Considering its relatively low expression in normal tissues, bringing potential safety and presumed tolerance in patients, thus CD276/B7-H3 becomes an attractive target for immunotherapy.

CAR-T therapy targeting CD276 both in vitro and in xenograft model demonstrated promising survival benefits [266]. Using antibody–drug conjugates to ablate CD276 + glioma cells simultaneously impaired tumor vescular [262], indicating a novel insight on the combination of anti-CD276 with anti-angiogenesis, which was supported when CD276 was confirmed to positively be related with VEGFA and MMP2 [267].

The immunotherapy value of CD276 has not been fully determined partially due to the un-defined isoforms, intracellular domain and ligands of CD276 [268]. Although such a type I transmembrane protein has almost 90% homologous amino acid sequence between human and murine [269], different and opposing outcomes were sometimes reported in murine tumor model compared with human tumor model [270], making the clinical trials in gliomas challenging.

#### CD47

CD47, also called integrin-associated protein (IAP) or MER6 [271], is ubiquitously expressed in astrocytoma cells as different isoforms [272, 273]. CD47 promotes GBM invasion and progression [274, 275], and also delivers a special “don’t eat me” signal by binding to signal-regulatory protein α (SIRPα or CD172a) on macrophages or DCs. Thus, anti-CD47 is suggested efficacious by promoting immune response ablating tumor cells via macrophage and/or DCs [276, 277], even through microglia [278].

Apart from integrins and SIRPγ (CD172b) [279], expression of SIRPα is also observed in brain tissues [280], astrocytomas [281]. SIRPα, also called CD172a, is vital to the “CD47-SIRPα axis”, which is more significant since SIRPα is only expressed on certain cells including myeloid cells and neurons leading to relatively high safety and efficacy.

In vitro experiment and murine model proved that anti-CD47 induces the M1-polarization of macrophages that promotes an immune active tumor microenvironment [282]. Hu5F9-G4, a humanized anti-CD47 antibody, manifested efficacy both in pediatric GBM and diffuse intrinsic pontine glioma cells [283]. Anti-CD47 was also proved to increase tumor-infiltrating CD8 + T cells that suppressed glioma cells and cancer stem cells [284]. SIRPα-Fc blocks CD47-SIRPα impressively, also triggering autophagy of glioma cells thus promoting survival in GBM models, and the prognosis is better with chloroquine [285]. Given that TMZ induced ER stress response in GBM then beneficial to phagocytosis, studies found that combination of TMZ and anti-CD47 therapy was with drastically improved efficacy in GBM model [286]. Since SIRPα polymorphism has become the obstacle to anti-CD47 therapy, other studies are urgently needed to elucidate the entire mechanism of CD47-SIRPα axis.

#### Cytokine therapy

Cytokines, produced by the immune microenvironment, could both be employed by tumors to suppress immune response and be employed by immune system to induce immune response [287]. Among the multiple cytokines, interleukins and interferons have been most widely used in cancer therapy with high efficiency.

IL-2 was first studied in glioma patients in 1986 [288], which the combined IL-2 and tumor vaccination was observed with remarkable side effects [289]. Notably, tumor responses were detected in 50% rGBM patients receiving the combined therapy of IL-2-encoding genes and herpes simplex virus type 1 thymidine kinase (HSV-TK) genes. In a phase I trial, HGG patients receiving the glioma cell vaccine admixed with IL-4-encoding genes transfected fibroblasts showed favorable clinical responses [290]. The safety of recombinant protein IL-13-PE38QQR was confirmed in a phase I trial [291]. In a subsequent phase III trial, IL-13-PE38QQR significantly increased PFS but not OS in rGBM patients (NCT00076986) [292].

In two phase II trials, TMZ with combined IFN-α exhibited improved efficacy in rGBM patients [293]. IFN-β also enhanced sensitivity to TMZ by inhibiting MGMT transcription preclinically [294, 295]. Besides, the combined IFN-β and standard chemoradiotherapy prolonged the survival of GBM patients in a phase I trial [296]. However, the combined IFN-γ and standard chemoradiotherapy failed to demonstrate clinical benefits in GBM patients [297, 298].

#### TAM therapy

Tumor-associated macrophages (TAMs), a significant component of tumor microenvironment, in glioma, are commonly defined as macrophages of peripheral origin and microglia, to regulate immune response and promote tumor progression [299, 300]. A recent study demonstrated that despite of inducing T cell and DC activation, neoadjuvant PD-1 blockade failed to overcome the immunosuppressive TAMs in rGBM, indicating the important role of TAM in resistance to treatment [301].

Previous study revealed immunosuppressive M2 macrophages populating TAM in glioma tissues, are associated with histological grade of glioma. Researchers also suggested macrophage colony-stimulating factor (M-CSF) is vital to shift of microglia/macrophage to M2 subtype, inducing tumor proliferation [302, 303]. Thus BLZ945, a CSF inhibitor, has been tested to target TAMs in mouse models of GBM with satisfactory survival with elimination of tumor cells and decrease of M2 in TAM [304]. Pyonteck et al. also pointed out GBM classification (proneural GBM in this case) and TAM phenotype rather than TAM number as a potential biomarker for anti-CSF therapy. Given that combination of PI3K and BLZ495 showed better OS, further clinical trials are needed [305]. PLX3397 is another efficacious CSF inhibitor in GBM models [306], however, the result of a phase II trial showed PLX3397 barely presented therapeutic effect (NCT01349036). Moreover, biomimetic tumor-on-a-chip on GBM have predicted promising outcome of co-targeting M2-TAM combined with anti-PD-1 [307]. In epithelioid GBM (with markers of the BRAF‐V600E and TERT C228T promoter mutations and the absence of IDH1 and IDH2 mutations), CSF-1R is also detected broadly on epithelioid GBM cells and combination of inhibiting BRAF-V600E and BLZ945 reduces cell viabilities [308]. Those recent studies in vitro indicate potential efficacy of targeting TAM with other immunotherapies.

As a lipophilic molecule, antibiotic minocycline could suppress the expression of microglial MMPs and attenuate the invasion of glioma [309], which minocycline could also be safely combined with radiation and bevacizumab [310]. Besides, cyclosporine A displayed efficacy in attenuating the survival and angiogenesis of glioma by inhibiting the infiltration of microglia [311]. Propentofylline was also proved to reduce tumor growth in GBM by directly targeting microglia [312].

#### Dendritic cell vaccine

DC vaccine (DCV), composed of powerful antigen-presenting cells (APCs), could induce effective immune responses.

In most of the clinical studies on DCVs, autologous tumor lysate and tumor-associated peptides were chosen as the antigen [313]. In two phase I trials (NCT00068510 NCT00612001), autologous tumor lysate (ATL)-pulsed DCV was proved with higher patient eligibility than glioma-associated antigen (GAA) peptide-pulsed DCV [314]. GBM6-AD/DC vaccine was well tolerated and induced immune response in rGBM patients [315](NCT01171469). In a phase I/II trial (NCT00766753), DVC with EphA2, IL-13Rα2, YKL-40, and gp100 as GAAs was also well tolerated and induced potent immune response, contributing to the progression free status in 9 out of 22 glioma patients [316]. In a phase I trial (NCT00576641), DVC with HER2, TRP-2, gp100, MAGE-1, IL-13Rα2, and AIM-2 as antigens significantly prolonged OS and PFS in newly diagnosed GBM patients [317]. In a subsequent phase II trial of the same DCV (NCT01280552), ICT-107, remarkable antitumor activity was observed and ICT-107-treated GBM patients presented improved PFS [318]. Immune responses and clinical benefits of GBM patients receiving DCV were reported in another phase II trial (NCT00576537) [319]. Tumor lysate-pulsed DCV in combination with standard chemoradiotherapy was proved feasible and safe in newly diagnosed GBM patients in two phase II trials (NCT01006044, NCT00323115) [320, 321].

Other studies explored the feasibility of mRNA-transfected DCV. The mRNA-transfected DCV was suggested to be safe, well-tolerated, and significantly prolonged the PFS of GBM patients by 2.9 times (NCT00846456) [322]. In addition, despite the increased Treg proportions, pp65-transfected DCV admixed with GM-CSF and TMZ significantly prolonged the PFS and OS of GBM patients [323].

## Low-grade glioma (LGG)

Notably, there was no significant difference in PFS and health-related quality of life of LGG patients receiving radiotherapy alone or temozolomide alone [16, 324]. Correspondingly, the combined radiotherapy and TMZ presented better clinical benefits [325, 326], with certain better prognosis when MGMT promoter methylation exists [327]. Hence, TMZ or radiotherapy as the single therapy for LGG is not recommended. Generally, most of the median overall survival of LGG patients are more than 10 years, only those with diffuse astrocytoma with IDH-WT are around 5 years [14, 328]. As discussed above, WHO grade I glioma, most of which is pilocytic astrocytoma, presents excellent prognosis, with reported 10 years overall survival as high as 100% after standard gross total resection [1, 14]. To WHO grade II glioma, 5 years survival rate of oligodendroglioma reaches 81%, while diffuse astrocytoma only reaches 50%.

### Alkylating agent

**Fig. 5:**
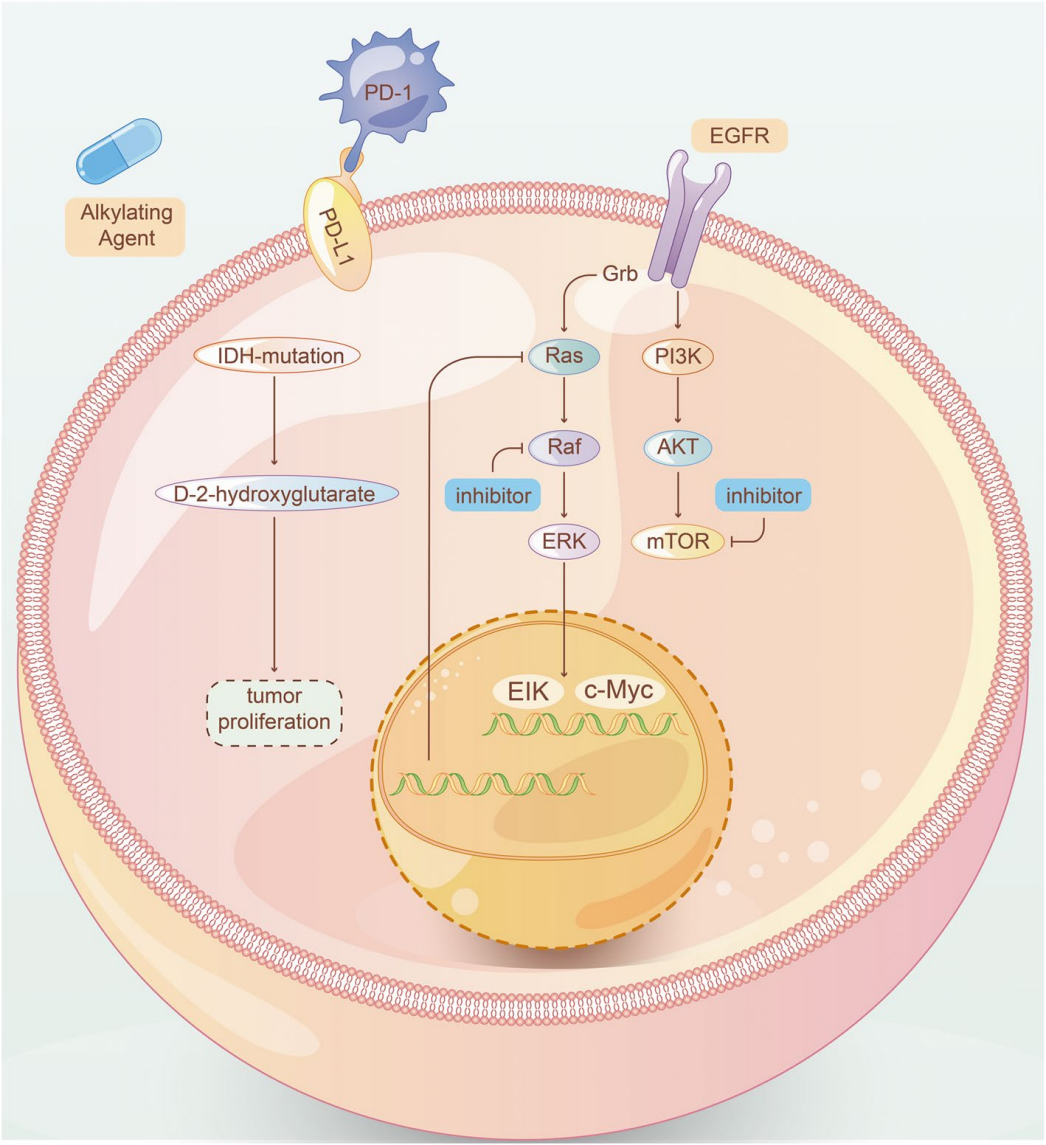
Candidate molecular targets amenable to targeted interventions in LGG

IDH mutation and consequently increased D-2-hydroxyglutarate level inhibit the expression of some DNA repair genes and anti-apoptotic proteins, such as MGMT, MLH3, RAD21 and SMC4 [329], which downregulates the intracellular glutathione level, apoptosis threshold of LGG and upregulates the sensitivity of LGG to alkylating agent [330] (Fig. 5, Table 2). Similarly, 1p/19q codeletion also correlates with the sensitivity of LGG to alkylating agent [331].

**Table 2:** Molecular targeted therapy of LGG

Reference& selected trials	Intervention	Design	Primary endpoint	Response	PFS	OS	Conclusions
Alkylating agent							
Baumert et al. 2016	standard radiotherapy/primary temozolomide	Randomized phase III, Open label	PFS		Median PFS (months) IDHmt/codel 62 IDHmt/non-codel 48 IDHwt 20		There is no significant difference between radiotherapy alone and TMZ alone
Reijneveld et al. 2016	radiotherapy/temozolomide	Randomized phase III Open label	PFS		Median PFS (months) RT alone 46 TMZ alone 39		The effect of temozolomide or radiotherapy on HRQOL or global cognitive functioning did not differ in LGG
Wahl et al. 2017	Adjuvant TMZ	Non-randomizes phase II	radiographic response rate	7 PR	Median PFS 3.8 years	Median OS 9.7 years	TMZ is beneficial as adjuvant therapy
Fisher et al. 2020	RT, TMZ, post-RT TMZ	Single arm Phase II	OS		Median PFS 4.5 years	3-year OS rate 73.5% 5-year OS rate 60.9%	Combination of TMZ and RT is better than RT alone
Ras/Mek/Erk							
Karajannis et al. 2014	Sorafenib	Non-randomized Phase II Open label					Sorafenib produced unexpected and unprecedented acceleration of tumor growth
Fangusaro et al. 2019	selumetinib	Non-randomized Phase II Open label	ORR	Stratum 1: 9 PR and 9 SD Stratum 2: 10 PR and 15 SD	2-year PFS Stratum 1: 70% Stratum 2: 96%		Selumetinib is active against BRAF aberrations and NF-1 associated pLGG
Hargrave et al. 2019	Dabrafenib	Single arm phase I/IIa Open label	ORR	1 CR and 13 PR	Median PFS 35.0 months		Dabrafenib is active
Perreault et al. 2019	Trametinib	Non-randomized Phase II Open label	ORR (primary objective)				
PI3K/AKT/mTOR							
Ullrich et al. 2020	Everolimus	Non-randomized Phase II	PFS at 48 weeks	1 CR and 3 PR(3D/volumetric analysis)			Everolimus is active against NF-1 associated patients
Wahl et al. 2017	Everolimus	Non-randomized Phase II	PFS-6		PFS-6 Grade II 84% Grade III/IV 55% Median PFS (years) Grade II 1.4 Grade III/IV 0.6	Median OS(years) Grade II not reached Grade III 2.9	Everolimus leads to disease stability

### Tyrosine kinase receptor pathway

#### tyrosine kinase receptor

Erlotinib, a kind of EGFR inhibitor, was mildly effective combined with rapamycin in pediatric LGG (pLGG), and disease stability was observed especially in patients with neurofibromatosis type 1 (NF1) [332]. Erdafitinib, a kind of FGFR inhibitor, was tested in LGG patients in a phase II clinical trial (NCT03210714).

#### Ras/Mek/Erk pathway

Abnormal activation of Ras/Mek/Erk pathway is the most common and major cause of genetic/epigenetic alterations in LGG. Different LGG subtypes activate this pathway in distinct ways, inducing carcinogenesis and tumor progression. Therefore, inhibiting this pathway is becoming a promising treatment option.

##### BRAF mutation

BRAF–KIAA1549 fusion and BRAFV600E mutation are the most prevalent genetic alternation in pLGG that are being increasingly focused as the therapeutic targets.

Sorafenib is a multi-kinase inhibitor targeting BRAF, VEGFR, PDGFR, and c-kit, which unexpectedly promotes the proliferation of the tumor cells in low grade astrocytoma [333]. Dabrafenib, a selectively robust inhibitor of BRAFV600 was mildly effective in patients with BRAF V600–mutant pLGG (NCT01677741) [334]. MEK inhibitor, Selumetinib, prolonged the survival of LGG patients (NCT01089101) [335–337]. Another inhibitor of MEK, Trametinib, is still being tested in a phase II trial (NCT03363217) [338].

##### NF1

Neurofibromatosis type 1 is an essential autosomal dominant genetic disorder resulted from loss-of-function mutations in gene neurofibromatosis type 1 that encodes a negative regulator of Ras GTPases under physiological condition [339] and influences the MAPK signaling. The loss-of-function mutation is usually found in anaplastic astrocytoma [340], which NF1 subsequently leads to the diffuse or pilocytic phenotype of pLGG [341]. When treated with carboplatin and vincristine, LGG patients with NF1 experienced prolonged PFS, OS and decreased toxicity [342]. Selumetinib was proved with high effectiveness in pLGG patients with NF1 [336]. A phase II trial exploring double-strain RNA as Toll-like receptor-3 agonist [343] to cure pLGG patients with NF1 is ongoing (NCT04544007). NCT03871257 and NCT04166409 are two simultaneous phase III studies investigating the efficacy of Selumetinib in LGG patients with or without NF1.

#### PI3K/AKT/mTOR pathway

Everolimus, the mTOR inhibitor, is promising and effective in recurrent/progressive NF1-associated LGG [344]. Nevertheless, for most LGG patients, Everolimus was only associated with a high degree of disease stability and unexpected tumor vascular alternations [345].

### IDH-mutation

Compared with children, IDH-mutation is more common in adult LGG. IDH-mutation was first detected from the exome sequencing of GBM [346]. Many follow-up studies proved that patients with IDH-mutation had better prognosis [347]. The mutant IDH1 with a neo-enzymatic activity could produce D-2-hydroxyglutarate whose accumulation in cells facilitates tumor proliferation and growth, increases the ROS level, and promotes hypermethylation in certain DNA sequence. As an inhibitor of IDH1, AG5198 suppressed proliferation of IDH-mutant tumor cells in animal model [348]. Inhibitors of D-2-hydroxyglutaratethat is the production of mutant IDH1 is under explored as a novel treatment for LGG (NCT03343197). Nevertheless, IDH-mutation as a significant early mutation site of glioma has also been proposed to no longer regulates tumor proliferation and invasion after tumor formation [349], So, the prospect of IDH-mutation as a therapeutic target in LGG remains controversial.

### PD-1

PD-1 is widely detected among LGG patients, but most of their tumor tissue are positive with PD-1 in a small proportion (< 5%). In some cases, over 50% cells were detected with PD-1 positive [350], which may be related to the methylation of PD-1 promoter in patients with LGG [351]. The expression of PD-1 by immune infiltrating cells in LGG indicates immune escape, while the methylation of PD-1 promoter indicates better prognosis of LGG. Besides, the expression of PD-1 may facilitate adjuvant therapy in patients with radiotherapy tolerance [352].

## Conclusion

Due to its relatively lower tumor malignancy, better prognosis, and higher chemotherapy sensitivity brought by IDH-mutation, researches for therapy of LGG are limited. However, some high-risk LGGs incompletely cured by surgical resection are prone to relapse and turn into high-grade gliomas with malignant and aggressive characteristics, which more postoperative adjuvant treatment modalities are necessary. The most common mutation in LGG is the abnormal activation of the Raf/MEK/Erk pathway. Therefore, there are many target inhibitors of this pathway and most of them are very effective. Different LGG subtypes have different genetic/epigenetic alterations, such as amplification and/or rearrangement of MYB/MYBL1, 1p/19q co-deletion, ATRX, and CDKN2A loss. These molecular changes are valuable prognosis signal and/or potential targets for the treatment of LGG. For instance, 1p/19q co-deletion is beneficial for LGG sensitivity to alkylating agent. Additionally, early enough resection combined with chemotherapy or adjuvant around surgery if necessary is gradually advocated instead of MRI every 3–6 months and resection only after tumor progression. As a majority of chemotherapy of LGG are carried out after surgical operation, instant molecular diagnosis is necessary in order to provide prognostic evidence and more precise target therapy. Combination of alkylating agent and inhibitor against Ras/Mek/Erk pathway is worthy of attempting.

For newly diagnosed GBM, the current standard therapy is alkylating agent chemotherapy combined with radiotherapy. Owing to the fact that standard therapy is limited to patients with MGMT promoter hypermethylation and there is no standard therapy for patients with rGBM, more effective target treatment modalities are urgently needed. In recent years, researchers have mostly adopted the scheme of combining therapies targeting different pathways in GBM. However, the therapy efficacy is often unsatisfactory mainly due to the existence of BBB, the complexity of tumor microenvironment, the heterogeneity of tumor tissues, and the tolerance of drugs. Therefore, some potential attempts could be made: 1) developing more effective drug delivery system to cross BBB, such as nanoemulsion for nasal administration or direct intracranial administration, 2) identifying and elucidating more complicated pathways such as PI3K for development of novel drugs 3) more timely and precisely molecular diagnosis of tumor cells [353]. With the publishing of WHO CNS5, gene and protein nomenclature is formally recommended and proved to be more effective and beneficial to clinic. And CNS5 has listed newly-discovered types of gliomas recently, also with a method of grading within types and combining histological and molecular grading, pointing out a legible way to diagnosis and associated treatment. Additionally, studies have confirmed few prognostic or (and) predictive biomarkers remain steady between GBM at the time of diagnosis and relapse because of the evolution of tumor. For instance, loss of expression of MSH6, a mutation of mismatch repair gene, is more frequently found among relapsed GBM than newly diagnosed GBM as a result of standard chemoradiotherapy (Stupp protocol), with mild decrease of MGMT methylation in recurrence as well [8]. Thus combination of molecular diagnostics and precise therapy is supposed to be executed both before and after a defined course of treatment. Furthermore, since Shepherd et al. discovered the elimination of the NTRK2-fusion-harboring cells by Larotrectinib promoted the predominance of untargeted subclone at relapse [95], it is presumed this certain alteration of molecular target might occur much more commonly in recurrence GBM, no matter which treatment we implement, indicating potential obstacles on future glioma therapy. Therefore, targeted therapies are indeed ideal weapons for precision and personalized medicine based on the detection of biomarkers throughout the management of glioma patients.

Moreover, immunotherapy has been an emerging field of tumor therapy. Various immune checkpoints inhibitors have performed well in several cancers. PD-1/PD-L1, CTLA4, TIM3 and other classic checkpoints have made remarkable progress in both pre-clinic and clinical trials. Given the complexity of tumor microenvironment and regulation of immune response, combination therapy is proposed, especially when the combination of anti-PD-1 and anti-CTLA4 presents promising efficacy on rGBM. Classic anti-PD-1 could be combined with various targets such as TIM3 and BTLA. Based on the ATP-ADO axis discussed above, combination of CD39 and CD73 and further exploration on regulation of local ADO concentration are also worth pursuing. Similarly, it is appealing to combine immunotherapy and other targetable pathway. Since CD276/B7-H3 is confirmed associated with angiogenesis, trials on anti-CD276 combined with Bevacizumab is attractive. EGFRvIII mutation leads to the unique extracellular domain of EGFR, becoming an ideal specific antigen for both vaccine and CAR-T therapy. As discussed above, given that single-target therapy induces recurrence and subsequently resistance to original treatment due to molecular heterogeneity and evolution of tumor, implementation of targeting multiple antigens or with antagonism of immunosuppressive cytokines is recommended. In short, more laboratorial and clinical effort is required when it comes to combination therapy (Table 3).

**Table 3:** Outlook

Pathways or targets	Limitations	Hotspots
EGFR	EGFR inhibitors or antibody appear inactive, partly due to the existence of BBB	Target on EGFR amplification and EGFRvIII
PI3K/AKT/mTOR	Most of the drugs experience poorly tolerance, and the regulation of this pathway is far too complex	combine PI3K/SKT/mTOR inhibitors with other drugs
MET	There is still no effective kind of drugs	Combination of c-MET inhibitor and PI3K inhibitors due to their cooperation to drug resistance
FGFR	Population of patients that could gain benefit from this target is extraordinarily small	
BRAF	Mutations of this target are rare	BRAFv600E in GBM needs to be further studied
NTRK	The incidence of NTRK gene fusion seems to be very low in glioblastoma	NTRK fusion as a therapeutic target is active and molecular heterogeneity screening in the diagnosis of GBM is significant
pRB	Regulation of cell cycle and apoptosis is complex	
P53	Effort on promoting the refolding of mutant proteins into wild-type conformations meets failure	Inhibitors of MDM2/4 and Weel kinase
TERT	Though TERT mutation is commonly identified in GBM, it has not yet become the main pharmacological target for tumor therapy	Novel inhibitors need to be developed
proteasome		
TGF-β	The function of TGF β protein family is complex and the regulatory pathways are widely crossed	Combine TMA and TGF inhibitors
PD-1		Combine PD-1 and other immunotherapy target
LAG-3	there are few trials about LAG-3 inhibitors or antibodies involved in GBM therapy	
CTLA-4		CTLA-4 inhibitors combined with TMZ, anti-PD-1 or other drugs appear promising
IDO1		Enzymatic and non-enzymatic activity of IDO
CD73 and CD39		In tumor microenvironment, both CD73 and CD39 participate in regulation of ATP-adenosine axis
CD27-CD70		Combination of CD27 agonist and CD70 inhibitor
CD276	Un-defined isoforms and intracellular domain with unknown ligand	CD276 is correlated with angiogenesis
CD47	Polymorphism of SIRPα, ligand of CD47	Anti-CD47 promotes phagocytosis of glioma

## Data Availability

Not applicable
